# Diaphragmatic breathing combined with abdominal drawing-in maneuver for walking function in post-stroke patients: a randomized controlled study protocol

**DOI:** 10.1186/s13063-023-07690-6

**Published:** 2023-10-19

**Authors:** Jianqing Su, Yunrong Ding, Yanjun Cao, Zengqiao Zhang, Mengxue Sun, Yajuan Zhang, Kunpeng Li, Wu Wang

**Affiliations:** 1 Department of Neurological Rehabilitation, the Second Rehabilitation Hospital of Shanghai, Shanghai, China; 2 Department of Osteoarticular Rehabilitation, the Seventh People’s Hospital Affiliated to Shanghai University of Traditional Chinese Medicine, Shanghai, China; 3 Department of Massage, the Fourth People’s Hospital Affiliated to Tongji University, Shanghai, China; 4 Department of Massage, Shanghai Yueyang Integrated Traditional Chinese Medicine and Western Medicine Hospital, Shanghai, China; 5 School of Rehabilitation Science, Shanghai University of Traditional Chinese Medicine, Shanghai, 201203 China

**Keywords:** Walking function, Core stability, Stroke, Diaphragmatic breathing, ADIM

## Abstract

**Background:**

Patients with stroke frequently experience walking dysfunction. Core training can help improve balance and walking function in patients with stroke. However, core training movements in clinical practice are numerous and differently targeted. Therefore, this study will investigate the improvement of walking function in patients with combined diaphragmatic breathing maneuver (DBM) and draw-in breathing technique (ADIM) training.

**Methods:**

This single-blind, randomized controlled preliminary will analyze the viability of DBM combined ADIM training versus routine rehabilitation therapy in patients with stroke with early to mid-stroke. Patients will be randomly assigned to either the DBM and ADIM training or the routine rehabilitation training. We will recruit 42 stroke inpatients from the Second Rehabilitation Hospital of Shanghai who meet the trial criteria and measure the balance and walking functions and improvement of that after 4 weeks of intervention. The primary outcome is the 10 m maximum walking test (10MWT). The secondary outcomes indices include the limits of stability test (LOS), Berg balance scale test (BBS), Functional Ambulation Categories test (FAC), Timed Up and Go test (TUG), trunk impairment scale test (TIS), ultrasound indicators of the diaphragm and transversus abdominis (UI), rhythmic weight shift test (RWS), walk across test (WA), Fugl-Meyer assessment of lower extremity (FMA-LE), and Barthel index of ADL test.

**Discussion:**

The primary objective of this project was to investigate the effects of DBM combined with ADIM on balance capacity and walking function for patients with early to mid-stroke. The outcomes of this study will hold significant implications for future clinical applications in rehabilitation.

**Trial registration:**

Chinese Clinical Trial Registry (ChiCTR), ID: ChiCTR2100054897. Registered on 28 December 2021.

**Supplementary Information:**

The online version contains supplementary material available at 10.1186/s13063-023-07690-6.

## Administrative information

**Table:** 

Title {1}	Diaphragmatic breathing combined with abdominal drawing-in maneuver for walking function in post-stroke patients: a randomized controlled study protocol
Trial registration {2a and 2b}	Registration number: ChiCTR2100054897
Protocol version {3}	The trial has been registered at the Chinese Clinical Trial Registry (ChiCTR) on December 28, 2021. No modifications have been made.
Funding {4}	This study was supported by the Youth Programs of Shanghai Municipal Health Care Commission [Grant number: 20214Y0321].
Author details {5a}	Jianqing Su, Yunrong Ding, Yanjun Cao, Zengqiao Zhang, Mengxue Sun, Yajuan Zhang, Kunpeng Li, Wu Wang. SJ: In charge of this research; SJ and DY: drafted manuscripts; CY, ZZ, SM and ZY: Investigation, Data curation; LK and WW: Conceives and designs. All authors know and agree with the final manuscript.
Name and contact information for the trial sponsor {5b}	Trial Sponsor: Shanghai Municipal Health Care Commission Address: No.300 Building 4, Shibocun Avenue, Shanghai Telephone: 23111111 Email: webmaster@wsjkw.sh.gov.cn
Role of sponsor {5c}	The funding resource has no part in the designing of this research and will have no part in the conduct of the research, the interpretation of the analysis, the interpretation of the data or the presentation of the results.

## Introduction

### Background and rationale {6a}

Stroke is an acute neurovascular disorder caused by inadequate blood supply to the brain; it has the characteristics of long duration, wide range, and severe degree. These conditions can dramatically limit a patient’s quality of life (QoL) and ability to manage activities of daily living (ADL) [1]. It has become the second leading cause of death and the third leading cause of disability worldwide, with an estimated incidence of 25% over a lifetime [2]. In recent years, with the acceleration of rescue speed and the improvement of thrombolysis technology, the mortality rate of patients with stroke decreased significantly. However, due to the advanced age of the patients and the uneven development between rehabilitation medicine and clinical medicine, the recovery process for central nervous system injuries tends to be protracted. Stroke has become the most common cause of long-term disability in adults [3]. While 50–70% of stroke patients regain functional independence, 15–30% will experience permanent disability, contributing significantly to social and economic burden by 2020 [4]. The World Health Organization speculates that about 75% of the lives of people with disabilities worldwide are related to the benefits of rehabilitation, so stroke rehabilitation requires urgent international attention [2].

Walking, as a fundamental activity of daily living (ADL), plays a pivotal role in the well-being and overall health of older individuals. However, following an early stroke, a study found that 63% of patients were unable to walk independently, and even after clinical and rehabilitation efforts, 22% remained unable to walk [5]. This disease severely restricts patients’ daily activities and social participation, greatly impacting their quality of life. Existing research predominantly focuses on rehabilitating lower limb function in hemiplegic patients, neglecting the importance of trunk control in walking recovery. However, as early as 1992, Wagenaar et al. began to observe the relationship between trunk and gait in patients with stroke [6]. By 2002, studies have shown that trunk control is related to walking function parameters such as walking speed, walking distance, and balance [7]. In 2006, Verheyden et al. evaluated trunk function with a trunk control test, trunk injury scale, Tinetti balance and gait subscale, functional walking classification, 10-m walking test, timing take-off test, and functional independence exercise scale. It was found that trunk function was significantly correlated with balance, gait, and walking ability [8]. Further investigations highlighted the influential role of core muscle strength on balance and walking function [9]. In recent years, several investigations have revealed that incorporating core stability training or respiratory training in interventions can enhance the activation of core muscles, promote trunk stability, and subsequently improve lower limb movement control, walking speed, and other aspects of ambulatory function [10, 11]. There exists a strong correlation between trunk stability and human ambulatory function. Thus, for the impairment of walking function, the treatment of core muscles cannot be ignored.

Currently, the training of core strength for patients with stroke mainly includes central intervention techniques based on repetitive transcranial magnetic stimulation (rTMS) and peripheral intervention training based on Bobath methods and core stability training. However, the high cost and difficulty in locating the core muscles make rTMS inaccessible for widespread use in rehabilitation institutions. Additionally, the time-consuming nature of various clinical core strength training methods often leads to patient fatigue. In addition, there is a need for a rehabilitation technique that requires less time and provides precise targeting of core stability. Furthermore, to investigate that, we want to accurately measure the change in core muscles and evaluate the parameters related to trunk balance and walking function after intervention.

Diaphragmatic breathing maneuver (DBM) is a slow, deep, and regular breathing training that consciously extends the time of inhalation and exhalation and focuses on abdominal ups and downs [12]. In recent years, regarding the effect of DBM on the walking function of patients with stroke, most studies mainly focused on walking endurance [13]. It is worth noting that DBM can effectively stimulate the core muscles by activating diaphragmatic muscle contraction, increasing intra-abdominal pressure, achieving the effect of stabilizing the spine, and improving the core stability of patients with stroke [14].

The draw-in breathing technique, also known as the abdominal drawing-in maneuver (ADIM), is a method that specifically engages the transversus abdominis muscle before activating other abdominal muscles. This technique plays a crucial role in stabilizing spinal segments. It is commonly used to enhance the lumbar spine and pelvic stability by restoring transversus abdominis and internal abdominal obliques. In 2015, Lee et al. provided the first evidence to highlight the positive effects of ADIM training on core and postural stability in non-athletes with core instability [15]. Since then, research into the effects of ADIM on trunk stability has begun to receive attention. Research has indicated that incorporating ADIM movements into bridging exercises leads to the enhanced strengthening of the abdominal muscles and improved trunk stability compared to performing bridging exercises alone [16]. Moreover, ADIM training has been found to improve gait parameters and reduce pain symptoms in patients with lower back pain, resulting in pain reduction and improved gait symmetry [17]. These suggest that ADIM can improve trunk stability and walking function. In recent years, ADIM combined with bridging/pelvic training is effective in improving trunk stability in patients with stroke; however, the benefits of ADIM in improving walking function in patients with stroke are unclear [18, 19].

### Objectives {7}

DBM and ADIM target different trunk muscle groups, but the main trunk muscle groups activated by the two exercises are complementary, and it is not known whether DBM combined with ADIM can improve trunk stability and thus further promote walking function. It is therefore important to identify the site of action of the technique on the trunk, to investigate the mechanism of its effect on trunk stability in patients with stroke, and to observe the effect of the technique on further walking function.

### Trial design {8}

To evaluate the efficacy of combining DBM with ADIM in patients with stroke, a randomized controlled trial utilizing a parallel design and employing an assessor-blinded methodology will be conducted. Participants are all inpatients of the Neurorehabilitation Department of Shanghai Second Rehabilitation Hospital who correspond with the inclusion criteria. The research projects are registered with the Chinese Clinical Trials Registry (ChiCTR-2100054897), and the study has been approved by the Ethics Committee of the Shanghai Second Rehabilitation Hospital (Shanghai, China). All participants will be informed of the trial and will sign a written informed consent form.

Patients who meet the inclusion criteria will be randomly grouped after basic data collection. This will be done by using a computer-generated random number table to randomly divide the subjects into a control group (receiving 4 weeks of conventional rehabilitation without other specific exercise interventions), and a study group (receiving 4 weeks of DBM + ADIM intervention alongside conventional rehabilitation) in a 1:1 ratio. Random numbers will be placed in opaque envelopes by independent persons not involved in this experiment to achieve allocation concealment. As this study uses an exercise intervention, it will be difficult to conceal the grouping from the therapist and subjects, but it will be possible to blind the outcome indicator measurer. We will arrange for a new assessor to replace the assessor familiar with allocation to achieve single blinding.

**Fig. 1:**
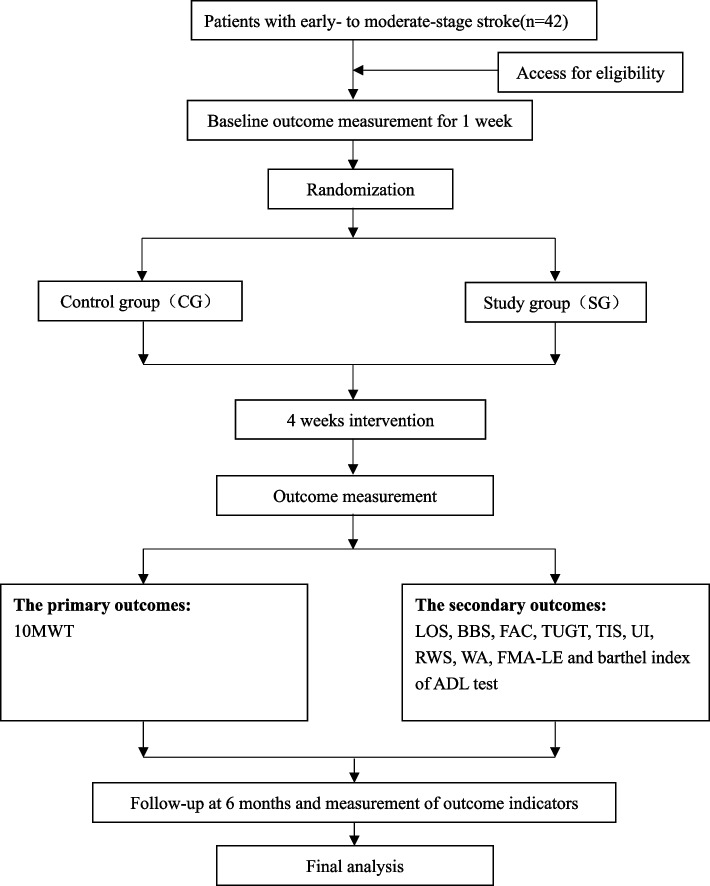
Flow chart of the study

**Fig. 2:**
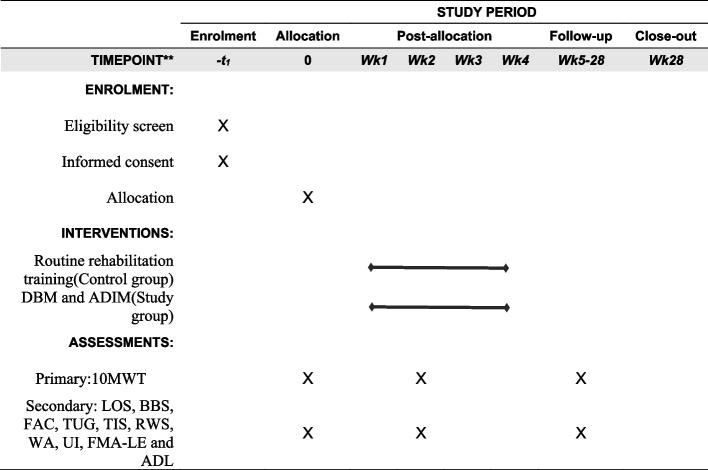
The schedule of enrolment, interventions, and assessments

In this trial, a superiority trial framework has been established to examine a specific core training approach, with the aim of improving walking function in patients with stroke. The trial process includes an initial assessment one week before the start date, a final assessment post-trial, and a follow-up evaluation six months later. These evaluations serve the purpose of measuring treatment effectiveness, assessing potential side effects, and documenting relevant baseline data and clinical information of the participants. The study procedure is shown in Fig. 1 and the period of the study in Fig. 2. The program follows the principles of SPIRIT 2013.

## Methods: participants, interventions, and outcomes

### Study setting {9}

All subjects will be recruited from the Second Rehabilitation Hospital of Shanghai. Treatment of patients will be carried out in the sports rehabilitation treatment unit of the hospital. Physiotherapists will provide rehabilitation treatment to all patients, including ADIM, DBM, and traditional rehabilitation, in strict accordance with the requirements of the trial and ethical standards.

### Eligibility criteria {10}

#### Inclusion criteria

1. Age between 40 and 85 years;
2. Consistent with a diagnosis of cerebral hemorrhage or cerebral infarction;
3. The level of Brunnstrom lower limb classification greater than III;
4. Course of disease 1 year;
5. Clear consciousness, no intellectual impairment, Modified Mini-Mental State Examination score (MMSE) more than 24;
6. Patients have signed an informed consent form.

#### Exclusion criteria

1. Patients with serious illness or in the acute stage of illness;
2. Those who with severe cognitive and verbal impairment or inability to cooperate;
3. Those who with Parkinson’s, spinal cord injury, or other conditions affecting trunk stability;
4. Those who with severe visceral organ disease;
5. Those who with specialized sports training.

### Who will take informed consent? {26a}

Trained research nurses will conduct the trial introduction, utilizing a comprehensive video presentation to highlight the key aspects of the study. In addition, patients will receive informative sheets. Research nurses will provide detailed discussions on the trial with patients, based on the information presented in the video and sheets. Thereafter, patients will be facilitated for an enlightened engagement with the attending consultant. Written consents from the patients choosing to participate in the trial will be obtained by the research nurses. Subjects will be given informed consent prior to the trial being conducted and written documentation will be shown in the Supplementary material.

### Additional consent provisions for collection and use of participant data and biological specimens {26b}

The study was carried out with full protection of subject rights and medical ethics in mind. Its main aim was to observe the improvement of patients’ walking ability through core training. None of the test indicators necessitated the collection of specimens from the subjects. In line with legal and ethical obligations, the trial will be overseen by independent entities such as the trial steering committees and data monitoring committees, ensuring the protection of subjects’ rights throughout the process. The arbitrary collection and utilization of subject data for other ancillary studies can complicate trial implementation and give rise to ethical and legal issues. Given this, there is no need for the collection and usage of subject data and biospecimens for other ancillary studies in this trial.

## Interventions

### Explanation for the choice of comparators {6b}

Physiotherapy for patients with stable early to mid-stroke vital signs who in keeping with the inclusion criteria, with reference to the routine rehabilitation program developed by the therapist in the *2017 edition of the Chinese Guidelines for Early Stroke Rehabilitation* and the *2019 edition of the Chinese Clinical Management Guidelines for Cerebrovascular Diseases (Abridged Version)—Stroke Rehabilitation Management*. The patients in the control group will undergo regular rehabilitation as described in item 11a.

### Intervention description {11a}

1. Control group (CG)
  Patients in the control group will receive routine rehabilitation training. Routine rehabilitation training will include (1) motor therapy and functional training for hemiplegic limbs; (2) sitting and standing balance training; (3) weight transfer training; (4) walking training; (5) and ADL training, among others. The therapist will inform the patient of the training prior to carrying out the rehabilitation. The duration of the regular rehabilitation will be 60 min five times a week for four weeks. All interventions for the control group will be carried out by three experienced intermediate therapists separately.
2. Study group (SG)
  In addition to receiving the same type of routine rehabilitation as the control patients, patients in the study group will also be required to undergo DBM and ADIM sessions. Each rehabilitation session for patients in the trial group will be divided into 40 min of regular rehabilitation and 20 min of DBM combined with ADIM training. The study group will complete 5 training sessions per week for 4 weeks in the same setting. All interventions for the study group will be delivered separately by three experienced intermediate therapists.

#### DBM training

Instruct the patient to lie in a supine position with the knees and hips in a slightly flexed and comfortable position. Instruct the patient to do a deep inhalation and consciously puff out the abdomen, pause briefly after a full inhalation, and hold this position for 10 s. This is followed by a slow exhalation with a conscious retraction of the abdomen on the exhalation, in a slow and deep rhythm. To ensure that the patient performs the exercise correctly, ask the patient to place one hand on the chest and the other on the abdomen. The patient is also advised to breathe deeply so that he or she only the hand on the abdomen is felt to be moving and not the hand on the chest. The DBM training is performed in 3 sets of 10 reps each and rests 1 min between sets. The workout is limited to 10 min.

#### ADIM training

Prior to the intervention, the therapist will receive a training session on the operation of ADIM training. The subject is positioned supine with the hips flexed at 40–60° and the knees flexed at 90–100°, with the arms resting on the trunk. Instruct the patient to delicately retract the navel towards the spine, maintaining a contracted abdomen while engaging in regular breathing. The successful contraction of the transversus abdominis muscle is defined as the sustained presence of progressive deep tension in the abdominal wall for a duration of 10 s. At this time the therapist can palpate the muscles on the medial aspect of the anterior superior iliac spine that are contracting. Patients who present with either of the compensatory patterns of posterior pelvic tilt, or downward heel pressure, will be considered non-compliant for this movement. The ADIM training is performed in 3 sets of 10 reps each and rests 1 min between sets. The workout is limited to 10 min.

### Criteria for discontinuing or modifying allocated interventions {11b}

The patient’s rehabilitation will be carried out under continuous and strict heart rate monitoring. The criterion for termination of exercise is an increase in the patient’s heart rate to more than 80% of their heart rate reserve. Furthermore, any adverse events such as dizziness, extreme dyspnea, or chest pain experienced by the patients will be promptly recorded by the therapist. These events will then be assessed by the researcher and specialist to determine whether they are exercise-related. If patients feel unwell during participation in the intervention, they will receive prompt and appropriate treatment.

### Strategies to improve adherence to interventions {11c}

1. Adequate pre-intervention education was provided to patients in the trial group.
2. The assessment of all patients will be completed within a week and will not take up too much time.

### Relevant concomitant care permitted or prohibited during the trial {11d}

In the “Eligibility criteria {10}” section, subjects cannot participate in other sports training programs during the trial.

### Provisions for post-trial care {30}

Details of the operation are in the “Criteria for discontinuing or modifying allocated interventions {11b}” section. Additionally, enrolment into the study ensures patients are duly protected against negligent harm through standard National Health Service (NHS) indemnity arrangements.

### Outcomes {12}

Participants will be assessed within 1 week of admission at baseline and the end of 4 weeks. The primary outcome is the 10 m maximum walking test (10MWT). Secondary outcome measures include the limits of stability test (LOS), Berg balance scale test (BBS), Functional Ambulation Categories test (FAC), Timed Up and Go test (TUG), trunk impairment scale test (TIS), ultrasound indicators of the diaphragm and transversus abdominis (UI), rhythmic weight shift test (RWS), walk across test (WA), Fugl-Meyer assessment of lower extremity (FMA-LE), and Barthel index of ADL test.

### Participant timeline {13}

Details of the timeline are in Fig. 2.

### Sample size {14}

The study was a randomized controlled trial, with the 10 m maximum walking speed of the study participants as the main outcome indicator observed. The study was found by sample estimation with the G*Power 3.1 software, which, combined with previous studies, resulted in an effect size of 1.1327272, a power value of 0.9, and a significance level of *a* = 0.05, giving a sample size of 36 cases. Considering that there may be a 15% attrition rate during the trial, the required sample size was obtained. The final sample size was calculated to be 42 cases.

### Recruitment {15}

Following the proposed timeline, patient recruitment for this study will commence on September 1, 2022, and will continue until 1 year later or until the desired sample size is achieved. As this is a single-center clinical trial with a clear patient source, we will collect patients who meet the recruitment requirements from the hospital medical record system based on inclusion and exclusion criteria. To facilitate effective communication with patients, a comprehensive plan and methodology will be developed, which includes obtaining informed consent and conducting a video presentation explaining the study’s objectives and procedures. In addition, we will explain to subjects the advantages and disadvantages of the treatment and the relevant safety measures to be taken during the trial. Based on the recruitment criteria, the study team will initially estimate the likelihood of inclusion for each subject and, after the subject has signed the informed consent form, will carry out the relevant tests to confirm whether the subject conforms to the inclusion/exclusion criteria.

## Assignment of interventions: allocation

### Sequence generation {16a}

Details of the operation are in the “Trial design {8}” section.

### Concealment mechanism {16b}

Details of the operation are in the “Trial design {8}” section.

### Implementation {16c}

Details of the operation are in the “Trial design {8}” section.

## Assignment of interventions: blinding

### Who will be blinded {17a}

Details of the operation are in the “Trial design {8}” section.

### Procedure for unblinding if needed {17b}

If the following events occur during the trial, blinding will be lifted.:

1. If the safety monitoring committee or data monitoring committee identifies an evident safety concern based on trial data that necessitate modifications to the trial or control group, or discontinuation of the trial, appropriate action will be taken.
2. Immediate intervention will be warranted if a participant enrolled in the trial reports an apparent adverse reaction or side effect to the drug being tested.
3. The results of the primary endpoint indicator of the trial have been sufficiently demonstrated that continued blinding is not required.

## Data collection and management

### Plans for assessment and collection of outcomes {18a}

All information about the patient shall be recorded by the assessor staff in a true, accurate, and timely manner on the case report form (CRF). The data manager will handle the relevant data exclusively and maintain strict confidentiality of participants’ personal information. To ensure patient privacy, all data will be identified using the subject number. Data will not be discussed without the researcher’s permission. At the end of the course, the assessor should submit a CRF promptly and submit a summary of the trial as required. A monitor will be specified by the research center to review the completeness and precision of the CRF.

Significantly, ultrasonic testing represents a pioneering measurement technique within the realm of medical assessment, warranting comprehensive elucidation of its testing methodologies:

- Using a Siemens Acuson Sequoia 512 compound B/M ultrasound, a wide-bore high-frequency line array probe with a frequency of 12–17 MHz was selected to record the B-mode ultrasound images.

The participant assumes a supine position with the knees in a relaxed and extended state. To obtain an intercostal view, the transducer is positioned longitudinally along the anterior axillary line on both sides, approximately at the 9th intercostal space. Evaluation takes place while the subject engages in forceful breathing, specifically during maximal inspiration. Notably, the diaphragm exhibits a distinctive three-layer structure, comprising two hyperechoic lines of pleura and peritoneum, flanking a central hypoechoic muscular region. Measurements of diaphragmatic thickness are obtained at the conclusion of both maximal inspiration and expiration. To ensure accuracy, three images are acquired and subsequently averaged.

Measurement of transversus abdominis thickness entails positioning the participant in a supine posture, with the abdomen in a relaxed state and breathing maintained at a calm pace. To establish a central placement, point for the probe, the examiner creates a vertical line originating from the unilateral anterior superior iliac spine, intersected by a horizontal line drawn at the level of the umbilicus. Once the probe is appropriately positioned, adjustments are made to capture images encompassing the three distinct layers of the abdominal muscles. It is important to note that image acquisition will be performed both at the end of calm breathing and during the peak activation of the ADIM.

### Plans to promote participant retention and complete follow-up {18b}

The present study proposes several viable avenues for enhancing participant recruitment and bolstering follow-up rates. Additionally, the protocol entails the meticulous collection of outcome data for those individuals who deviate from the designated treatment regimen or withdraw from the study group altogether:

1. A transparent and easily comprehensible trial prospectus, accompanied by a comprehensive informed consent form, is imperative for any clinical trial. The trial prospectus must furnish an unambiguous and detailed account of the trial’s purpose, methodology, and rights of the participants, to ensure that the participants are privy to the full corpus of information related to the trial’s conduct. Furthermore, the informed consent form must explicate participant obligations and rights, while concurrently clarifying any ambiguities that may arise during the trial.
2. Friendly subject recruitment and screening process. During the recruitment and screening process, explain and ask questions in a friendly and welcoming manner to help subjects dispel their doubts and concerns while minimizing the cost of time and effort to the subjects.
3. The habitual persuasion of patients to undergo the intervention amidst the clinical trial.
4. Developing a rigorous and efficacious follow-up program.
5. Collection of outcome data after subjects have withdrawn or changed treatment regimens: If the patient has been receiving the intervention for more than 2 weeks, the patient’s data should still be notes and statistics.

### Data management {19}

During the test, what is recorded in the CRF should be true and accurate. The CRF must comply with the following criteria:

1. The data must be recorded in black marker and the assessor’s signature is required;
2. If the patient has been receiving the intervention for more than 2 weeks, the patient’s data should still be notes and statistics;
3. If there is an error in the record that needs to be rectified, the recorder should draw a horizontal line under the original record and then sign the revision with the date of the correction.

Note that it is important to ensure that the original record is identifiable after the correction is made.

### Confidentiality {27}

Details of the operation are in the “Plans for assessment and collection of outcomes {18a}” section.

### Plans for collection, laboratory evaluation, and storage of biological specimens for genetic or molecular analysis in this trial/future use {33}

As this study did not involve biological specimen collection, laboratory analysis, or storage, this item was not applicable to our trial.

## Statistical methods

### Statistical methods for primary and secondary outcomes {20a}

Statistical analysis of research data will be performed by health statisticians and major researchers using SPSS 24.0. The statistical tests will all be two-sided. The level of statistical significance will be set at 5%. The measured data will be presented as mean ± standard deviation. Paired *t*-tests will be used for within-group controls before and after the trial. Comparisons between the two groups will be made using *t*-tests or rank sum tests.

### Interim analyses {21b}

When 50% of patients have been randomized and completed 6 months of follow-up, the primary endpoint will undergo an interim analysis by a statistician and the results will be presented to the trial data monitoring committee (DMC). Based on the assessment by the DMC, the trial may be stopped or undergo appropriate modifications. Furthermore, the decision to terminate the trial will be made solely by the trial data monitoring committee (DMC) or the trial management team in the event of a serious adverse event, serious safety issue, inability to continue the trial, or failure to demonstrate a predetermined hypothesis.

### Methods for additional analyses (e.g., subgroup analyses) {20b}

In this trial, our primary objective was to assess the effectiveness of a novel core training approach for patients with stroke compared to a conventional training approach, using a simple comparative trial design to establish the superiority of one treatment group over the other. As the trial design is straightforward and robust, with no apparent confounding factors or subgroups, no additional subgroup or correction analysis is deemed necessary.

### Methods in analysis to handle protocol non-adherence and any statistical methods to handle missing data {20c}

If the patient has been receiving the intervention for more than 2 weeks, the patient’s data should still be recorded and counted.

### Plans to give access to the full protocol, participant level-data and statistical code {31c}

The results will be published in journals.

## Oversight and monitoring

### Composition of the coordinating center and trial steering committee {5d}

The trial steering committee, constituted of a cohort of seasoned professionals, assumes a critical obligation in safeguarding the scientific soundness, lucidity, and adherence to the clinical trial process. In the following sections, we delineate their compositional classification, functional allocation, and respective duties:

- 1. Our trial steering committee is made up of the following members:
  - A chairperson, who is a senior professional lead physician
  - Several medical experts or researchers, representing different subject areas, such as biostatistics and clinical practice
  - An ethics expert.

- 2. Role
  - To assess and review the design and conduct of clinical trials to ensure that they meet scientific ethical and regulatory requirements
  - Develop and review documents such as clinical trial protocols, study protocols, informed consent forms, etc.
  - Monitoring the progress and results of clinical trials
  - Giving professional advice and recommendations to assist in improving the quality and safety of trials.

- 3. The responsibilities of the trial steering committee include:
  - Ensuring the scientific validity and safety of clinical trials
  - Overseeing ethical issues in the trial process
  - Providing professional advice and opinions to assist investigators in resolving problems in trials
  - Protecting the rights and safety of trial participants.

### Composition of the data monitoring committee, its role and reporting structure {21a}

The data monitoring committee is a team of a range of professionals who monitor and assess the quality and integrity of data, data security, and confidentiality needs in clinical research. Its organizational structure and roles are as follows:

- 1. The organizational structure
  - A research data manager or a representative of the data management committee (DMC)
  - Statisticians or professional data analysts
  - Pharmacologists or medical specialists
  - Biostatisticians
  - Computer scientists or information technology specialists

- 2. The role of the data monitoring Committee includes:
  - Monitoring the integrity and quality of data in the study
  - Reviewing data collection, documentation on data processing and data analysis
  - Overseeing the processing and analysis of the data
  - Checking data to ensure compliance with the research plan and research protocol
  - Dealing with ethical and legal issues relating to data
  - Conducting data and security assessments and ensuring compliance with relevant regulations.
  - Protecting the security and confidentiality of data

### Adverse event reporting and harms {22}

Details of the operation are in the “Criteria for discontinuing or modifying allocated interventions {11b}” section.

### Frequency and plans for auditing trial conduct {23}

The appraisal of a trial’s execution is an essential aspect of the trial process. It includes protocol, interim, and end-of-trial reviews. The ethical committee and autonomous DMC scrutinize the trial protocol while the interim trial audits are examined on a yearly basis, and the end-of-trial audits are undertaken exclusively by the independent DMC. Adherence to trial protocol by the trial team, alongside the utilization of audit mechanisms and tools, are indispensable to ensure scientific integrity and precision of the trial outcomes.

### Plans for communicating important protocol amendments to relevant parties (e.g., trial participants, ethical committees) {25}

Any modifications to the study protocol that possess the potential to influence patient safety, the investigation’s execution, or the potential benefits bestowed upon patients, including adjustments to the research objectives, design, or significant intervention administration, will be subject to solicitation for evaluation and expert analysis. These evaluations will transpire through well-established channels of communication with pertinent personnel, whereby the suggestions and proposed alterations will be meticulously scrutinized. Any formal modifications to the protocol that adhere to the highest standards of clinical trial design and ensure the rationality and validity of the study protocol will be efficiently implemented.

### Dissemination plans {31a}

At the end of the trial, we will take the following steps to communicate the results of the trial:

1. Dissemination of trial findings to participants will be conducted via telephone at the end of the study period. Comprehensive explanations of the trial’s objectives, results, conclusions, and recommendations will be provided by the researchers during the telephone communication.
2. The communication of research outcomes to healthcare professionals will be facilitated through diverse means such as dissemination in reputable journals, presentation at scholarly conferences, and engagement in online forums and workshops.
3. In order to ensure scientific integrity and data rigor, trial findings are presented for evaluation to a data monitoring committee or an intellectual property protection agency. Publication of the results is only considered after receipt of constructive feedback and verification by the aforementioned entities.

## Discussion

The study expects to investigate the effects of DBM combined with ADIM on balance capacity, walking function, and quality of life in early to mid-stroke patients.

Patients with stroke often experience weakened trunk muscles, which leads to impaired balance, reduced ability to shift their center of gravity, and limited walking function. A previous study has shown a positive effect of enhanced core stability on walking function in patients with stroke [20]. Therefore, this current study aims to utilize DBM combined with ADIM training to activate the core muscles of the trunk, improve abdominal muscle strength, and enhance trunk stability in patients with stroke, thereby improving their balance and walking function and improving their quality of life.

In everyday life, the voluntary contraction of the human trunk muscle groups counteracts some of the instability of distal limb movements and unpredictable disturbances from the external environment. During locomotion or ambulation, the core musculature is sequentially activated prior to the activation of peripheral limb muscles, facilitating the smooth execution of non-vigorous movements [21]. Motor control of the trunk is highly influential in distal limb movement and is associated with functional movement in individuals [9]. The significance of core muscle strength in relation to maintaining body balance and walking function is clearly evidently.

The core muscles are concentrated in the trunk and lower limb areas of the body. It plays an important role in trunk stabilization, walking initiation, and fall prevention [22, 23]. The transversus abdominis muscle, a band-like structure situated within the abdominal region, extends across multiple joints and serves as a pivotal motor hub for the limbs and head within the core area. Its strength directly affects the movement of the distal limbs of the body [7]. Notably, combining breathing training with core training yields better results in improving trunk stability compared to single-factor interventions such as core training [24]. The diaphragm is a key muscle in the study of breathing training. In combination with the transversus abdominis, the diaphragm is effective in maintaining spinal stability through the adjustment of intra-abdominal pressure [25]. Based on these findings, this trial aims to investigate the effect of DBM combined with ADIM on the rehabilitation of patients with stroke in terms of balance and walking function.

Previous studies on core stroke training have predominantly relied on scales or instruments to evaluate patients’ balance or walking function. Although these assessments are effective in assessing patients’ function, they make it difficult to further explain the site of action of the intervention in scientific studies and are not conducive to an in-depth study of the mechanism of action of the treatment. In contrast, musculoskeletal ultrasound has the advantages of portability, dynamic observation, and no radiation and is simpler to perform than ultrasound in the diagnostic department [26, 27]. In recent years, there has been an increase in studies on the use of ultrasound to observe morphological changes in the diaphragm and transversus abdominis muscle [28]. According to recent reports, ultrasound can effectively reflect changes in diaphragm thickness and mobility [29]. In 2015, Kapping found that ultrasound observation of transversus abdominis thickness in the clinical setting had reliable reliability and validity through a rigorous trial comparison [26]. Based on the above, this study will use musculoskeletal ultrasound to observe and measure morphological changes in the diaphragm and transversus abdominis muscles before and after the trial, combined with scales for balance and walking function, to assess the effect of the intervention in both morphological and functional terms.

The 10MWT, a commonly used instrument for assessing ambulation in stroke survivors, plays a pivotal role in objectively measuring walking speed, gait stability, and overall restoration of walking function [12, 30]. Research has demonstrated that the 10MWT exhibits favorable reliability and validity, thereby providing dependable data to facilitate the monitoring and advancement of rehabilitation interventions [31]. Additionally, the high sensitivity of the 10MWT enables the identification of even subtle enhancements in ambulatory ability. Consequently, it serves as a crucial tool for tracking alterations in walking function specifically in patients with stroke. Given these considerations, we selected the 10MWT as the primary outcome measure for this study.

There are still some limitations to this study due to some objective reasons: (1) strict control of inpatients in hospitals due to the epidemic situation, with a relatively homogenous source of subjects; (2) the ADIM technique is slightly difficult to operate and subjects have difficulty understanding the treatment; (3) unlike medication, rehabilitation training necessitates technical expertise and therapist guidance, making it challenging to implement double-blind protocols.

To adequately address the aforementioned limitations and minimize experimental errors, this study will implement the following measures: (1) it is difficult to conduct multicenter studies under strict epidemiological control, and when collecting cases in our hospital, we try to collect patients with stroke with different causes that meet the inclusion criteria; (2) multiple pre-intervention educations on ADIM techniques for therapists and subjects; (3) to minimize potential bias in trial outcomes, distinct personnel will be assigned for trial randomization and outcome assessment, ensuring the maintenance of single blinding.

Patients with stroke often have reduced trunk stability due to a lack of core strength, which in turn impairs walking function. To address this issue, our trial aims to focus on activating two critical core muscle groups, namely the diaphragm and transversus abdominis, through a combination of DBM and ADIM. Based on this training, which increases abdominal pressure and improves trunk stability, this trial will further investigate the extent to which this training improves balance and walking function in patients with early to mid-stroke.

## Trial status

The trial was registered in December 2021 and is currently in the recruitment phase.

### Supplementary Information

**Additional file 1.**

## Data Availability

The process of intra-study data sharing shall be overseen by the data management coordinating center, with valuable contributions from the data management subcommittee. Access to the datasets shall be granted to all principal investigators.

## Abbreviations

ADLs: Activities of daily living
DBM: Diaphragmatic breathing maneuver
ADIM: Draw-in breathing technique
CG: Control group
SG: Study group
10MWT: 10 M maximum walking test
LOS: Limits of stability test
BBS: Berg balance scale test
FAC: Functional Ambulation Categories test
TUG: Timed Up and Go test
TIS: Trunk impairment scale test
UI: Ultrasound indicators of the diaphragm and transversus abdominis
RWS: Rhythmic weight shift test
WA: Walk across test
FMA-LE: Fugl-Meyer assessment of lower extremity
CRF: Case report form
