# Supplementary material for: Diaphragmatic breathing combined with abdominal drawing-in maneuver for walking function in post-stroke patients: a randomized controlled study protocol
Source: Trials. 2023 Oct 19;24:677. doi: 10.1186/s13063-023-07690-6 (PMC10588227; doi:10.1186/s13063-023-07690-6)
Supplement: Supplementary file 1 — Additional file 1. [file 13063_2023_7690_MOESM1_ESM.docx]

**Informed consent**

Dear Patient：

Your doctor has diagnosed you with a stroke and other symptoms____________. We are doing a study on the effect of abdominal breathing combined with draw-in training on walking function in patients with early- to moderate-stage stroke. The study has been reviewed by the Ethics Committee and consent has been given to proceed with the clinical study. Thank you for your understanding and support. Before you decide to take part in this study, please read the following carefully as it will help you to understand the study and how to conduct it.

**I. Background and purpose of the study.**

A randomized controlled trial of abdominal breathing combined with the draw-in training in patients with early- to moderate-stage stroke walking impairment was used to find a treatment for walking impairment after stroke and to improve the ability and quality of life of patients.

**II. Who is not suitable to participate in this experiment?**

a. Those who are participating in other relevant clinical trials

b. All patients who do not meet the inclusion criteria for this trial.

**III. What do you need to do if you take part in an experimental study?**

a. Before you participate in the study, your doctor will take and record your medical history and assess your balance and walking function with the Fugl-Meyer Motor Function Scale for the lower limbs, Berg Balance Scale (BBS), Functional Ambulation Scale (FAC), Trunk Impairment Scale (TIS), 10m Walk Timed Test, Timed Up and Go Test (TUG), Wisconsin Scale (WGS), diaphragm ultrasound, transversus abdominis ultrasound, ADL score and the American BanlanceManager Balance Test System.

b. If you volunteer, you will follow our treatment for a period of 4 weeks.

c. Please be assured that we will keep your personal information confidential.

d. If you have any questions about this subject, please feel free to ask them and we will answer them for you. You may choose to withdraw for special reasons after our assessment.

**IV. Signature of the informed consent.**

**Project Title:** Clinical study on the effect of abdominal breathing combined with draw-in training on walking function in patients with early- to moderate-stage stroke

**Consent Affirmation:** I have been informed of the above presentation and have had the opportunity to discuss and ask questions about this study with my doctor and the questions I have asked have been answered to my satisfaction. I am aware of the protocol and precautions for this study and I agree to participate in this study and am willing to sign the informed consent form.

ID number: ________________________

Name：________________________

Date：________________________
